# Phosphorescent Energy Downshifting for Diminishing Surface Recombination in Silicon Nanowire Solar Cells

**DOI:** 10.1038/s41598-018-35356-w

**Published:** 2018-11-19

**Authors:** Hyun-Tak Kim, Kangmin Lee, Wonjoo Jin, Han-Don Um, Minsoo Lee, Eunhye Hwang, Tae-Hyuk Kwon, Kwanyong Seo

**Affiliations:** 1 Department of Chemistry, Ulsan National Institute of Science and Technology (UNIST), Ulsan, Republic of Korea; 2 Department of Energy Engineering, Ulsan National Institute of Science and Technology (UNIST), Ulsan, Republic of Korea

## Abstract

Molecularly engineered Ir(III) complexes can transfer energy from short-wavelength photons (λ < 450 nm) to photons of longer wavelength (λ > 500 nm), which can enhance the otherwise low internal quantum efficiency (IQE) of crystalline Si (c-Si) nanowire solar cells (NWSCs) in the short-wavelength region. Herein, we demonstrate a phosphorescent energy downshifting system using Ir(III) complexes at short wavelengths (300–450 nm) to diminish the severe surface recombination that occurs in c-Si NWSCs. The developed Ir(III) complexes can be considered promising energy converters because they exhibit superior intrinsic properties such as a high quantum yield, a large Stokes shift, a long exciton diffusion length in crystalline film, and a reproducible synthetic procedure. Using the developed Ir(III) complexes, highly crystalline energy downshifting layers were fabricated by ultrasonic spray deposition to enhance the photoluminescence efficiency by increasing the radiative decay. With the optimized energy downshifting layer, our 1 cm^2^ c-Si NWSCs with Ir(III) complexes exhibited a higher IQE value for short-wavelength light (300–450 nm) compared with that of bare Si NWSCs without Ir(III) complexes, resulting in a notable increase in the short-circuit current density (from 34.4 mA·cm^−2^ to 36.5 mA·cm^−2^).

## Introduction

Solar cells are being developed in various forms, from crystalline silicon-based first-generation solar cells to thin-film-based second-generation solar cells, and organic-based third-generation solar cells^1^. In addition, new types of solar cells including organic/inorganic hybrids have recently been developed, such as perovskite solar cells, perovskite/silicon tandem solar cells, and Si/PEDOT:PSS solar cells^2–4^. Among the various types of solar cells, crystalline silicon (c-Si) solar cells compose over 90% of the current photovoltaics (PV) market because of their relatively high efficiency and stability compared with other types of solar cells^5^. However, c-Si has a high refractive index of 3.5–6, which means that more than 30% of the incident light is reflected by the surface of c-Si^6^. Therefore, much research has been conducted to reduce light reflection and improve light trapping within c-Si substrates^7–12^. Among various surface modification techniques, nanowire texturing on the c-Si surface is considered as a promising approach to improve the power conversion efficiency (PCE) of c-Si solar cells owing to the superior light trapping efficiency over a wide wavelength range from 300 to 1100 nm^13,14^. However, further improving the PCE of c-Si nanowire solar cells (NWSCs) is hampered due to severe surface recombination accelerated by the large surface area of the NW structure^15^. In particular, the worsening of surface recombination causes a dramatic decrease in the number of photons collected over the wavelength range from 300 to 450 nm, which was about 30% of the external quantum efficiency (EQE) values previously reported^16^. Accordingly, developing an efficient and reliable system to diminish surface recombination would be valuable for developing high-efficiency c-Si NWSCs.

Over the last decade, advanced luminescent materials exhibiting quantum cutting or energy downshifting have been developed. In these processes, high-energy photons are converted into lower-energy photons with radiative decay^17–19^. Among them, energy downshifting materials, which absorb short-wavelength photons and emit long-wavelength photons, is one way to diminish surface recombination. The energy downshifting phenomenon has been observed in rare-earth materials and some II−VI semiconductor nanoparticles, which are usually costly and toxic^20^. On the other hand, Si nanoparticles (Si NP) and graphene quantum dots (GQDs) have attracted significant attention as alternative energy downshifting materials because of their low cost and non-toxicity. However, like carbon quantum dots, the luminescent quantum yield (QY) of GQDs is usually low (7% to 11%) owing to their surface defects and different lateral size^21^. Therefore, it is still necessary to develop a novel types of energy downshifting material with reasonable molecular engineering strategy.

In this study, we designed four different iridium-based phosphorescent energy downshifting materials for application in c-Si NWSCs; c-Si NWSCs generally have a low EQE in the short-wavelength region (300–450 nm), which matches well with the absorption range of Ir(III) complexes. Compared to conventional quantum-dot-based energy downshifting systems^20–23^, the triplet-singlet relaxation based phosphorescent system has several advantages, such as tremendous molecular engineering strategies, large Stokes shift preventing self-quenching, a long exciton diffusion length in crystalline film, and reproducible synthetic processes^24–28^. In addition, Ir(III) complexes exhibit additional advantages, such as high QY and easy control of the energy levels via ligand exchange. The long exciton diffusion lengths of these complexes, resulting from their long exciton lifetimes, would increase the energy downshifting efficiency. Furthermore, Ir(III) complex layers were applied reproducibly to fabricate c-Si NWSCs via an ultrasonic spray deposition (USD) method, which enabled the formation of highly crystalline and uniform energy downshifting layers. The high crystallinity of the Ir(III) complex layer formed via USD would enhance the energy downshifting efficiency of the c-Si NWSCs compared to the amorphous layer, because of the greatly suppressed non-radiative decay from exciton–optical phonon coupling^29^. The c-Si NWSCs with Ir(III) complexes showed EQE values which were twice those of bare c-Si NWSCs at short wavelengths, and an improved PCE (16.4%) compared with that of a reference device without the Ir(III) complex layer (15.5%). These results indicate that the energy downshifting effect of the Ir(III) complex was effectively applied in the c-Si NWSCs. Hence, we expect this work to open up a new approach for overcoming the short-wavelength photon induced poor carrier collection of c-Si NWSCs via unique phosphorescent energy downshifting systems.

## Results

### Synthesis and optical properties of Ir(III) complexes

**Figure 1:**
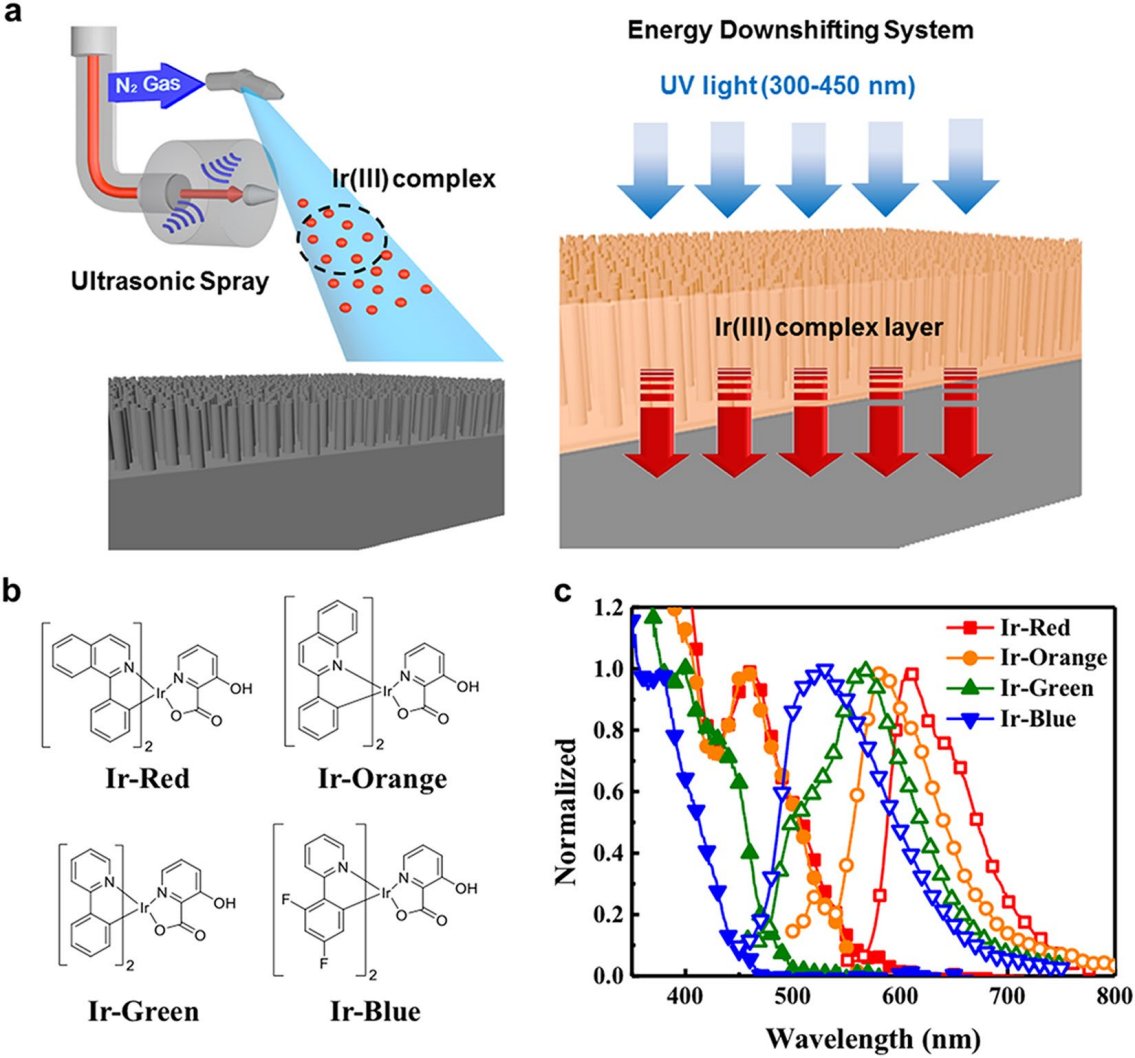
Phosphorescent energy downshifting system in c-Si NWSCs. (**a**) Schematic of the USD coating process and energy downshifting from an Ir(III) complex to c-Si NWSCs. (**b**) Chemical structures of Ir-Red, Ir-Orange, Ir-Green, and Ir-Blue. (**c**) Absorption (solid symbols; red squares for Ir-Red, orange circles for Ir-Orange, green upward triangles for Ir-Green, and blue downward triangles for Ir-Blue) and PL (open symbols) spectra of USD-coated Ir(III) complex films.

Figure 1a shows a schematic illustration of the USD coating process for an energy downshifting layer, as well as a plausible working mechanism for the energy downshifting layer in c-Si NWSCs. The USD process created uniform and highly crystalline energy downshifting layers on the c-Si NW surface. The Ir(III) complex-based energy downshifting layers convert ultraviolet light into visible light through the process of triplet based phosphorescence, resulting in a reduction of surface recombination and enhancement of the light absorption capability of the device in short wavelength region (Scheme S1). Four different iridium-based energy downshifting materials with different metal-to-ligand charge-transfer (MLCT) absorption maxima (350–500 nm) were designed for application in c-Si NWSCs; the surface recombination of c-Si NWSCs can be diminished for by tuning the absorption range of the Ir(III) complexes. More detailed synthetic processes are shown in Scheme S2^26^. To control the absorption and emission characteristics of the Ir(III) complexes, four different hydrophobic primary ligands (Fig. 1b) were used: 1-phenylisoquinoline (1pq), 2-phenylquinoline (2pq), phenylpyridine (ppy), and difluorophenylpyridine (F_2_ppy). These ligands have been shown to afford high-QY materials when incorporated with iridium^26,30,31^, which emit light of different colors ranging from blue (Ir-Blue), green (Ir-Green), and orange (Ir-Orange) to red (Ir-Red) with increasing conjugation length (Fig. 1b).

Figure 1c shows the absorption and emission properties of the developed Ir(III) complex-based thin films. The absorption spectra of the Ir(III) complex films indicate a π-π* ligand-centered transition at wavelengths shorter than 400 nm. Ir-Blue, Ir-Green, Ir-Orange, and Ir-Red exhibited MLCT at 376, 400, 455, and 462 nm, and the corresponding maximum emission peaks (*λ*_*max*_) appeared at 528, 562, 583, and 604 nm, respectively. All the Ir(III) complexes exhibited large Stokes shifts of over 100 nm, which would prevent self-quenching^32^. The QY values of the Ir(III) complex films was measured by integrated sphere spectrofluorometer, followed the order: Ir-Orange (10.4%) > Ir-Blue (8.1%) > Ir-Red (5.9%) > Ir-Green (5.4%). All emission wavelengths of the Ir(III) complex films are red-shifted compared to their solution states (Supplementary Fig. 1). The energy levels of the highest occupied molecular orbitals (HOMO: −5.73 to −5.44 eV) and lowest unoccupied molecular orbitals (LUMO: −3.19 to −2.94 eV) of the Ir(III) complexes included the energy bands of silicon (conduction band: −4.05 eV), valence band: −5.15 eV), as shown in Supplementary Fig. S2. These results indicate that the energy band structures of the Ir(III) complexes satisfy the condition of long-range energy transfer from the Ir(III) complexes to c-Si NWSC^24^.

### Spectroscopic properties of energy downshifting effect

**Figure 2:**
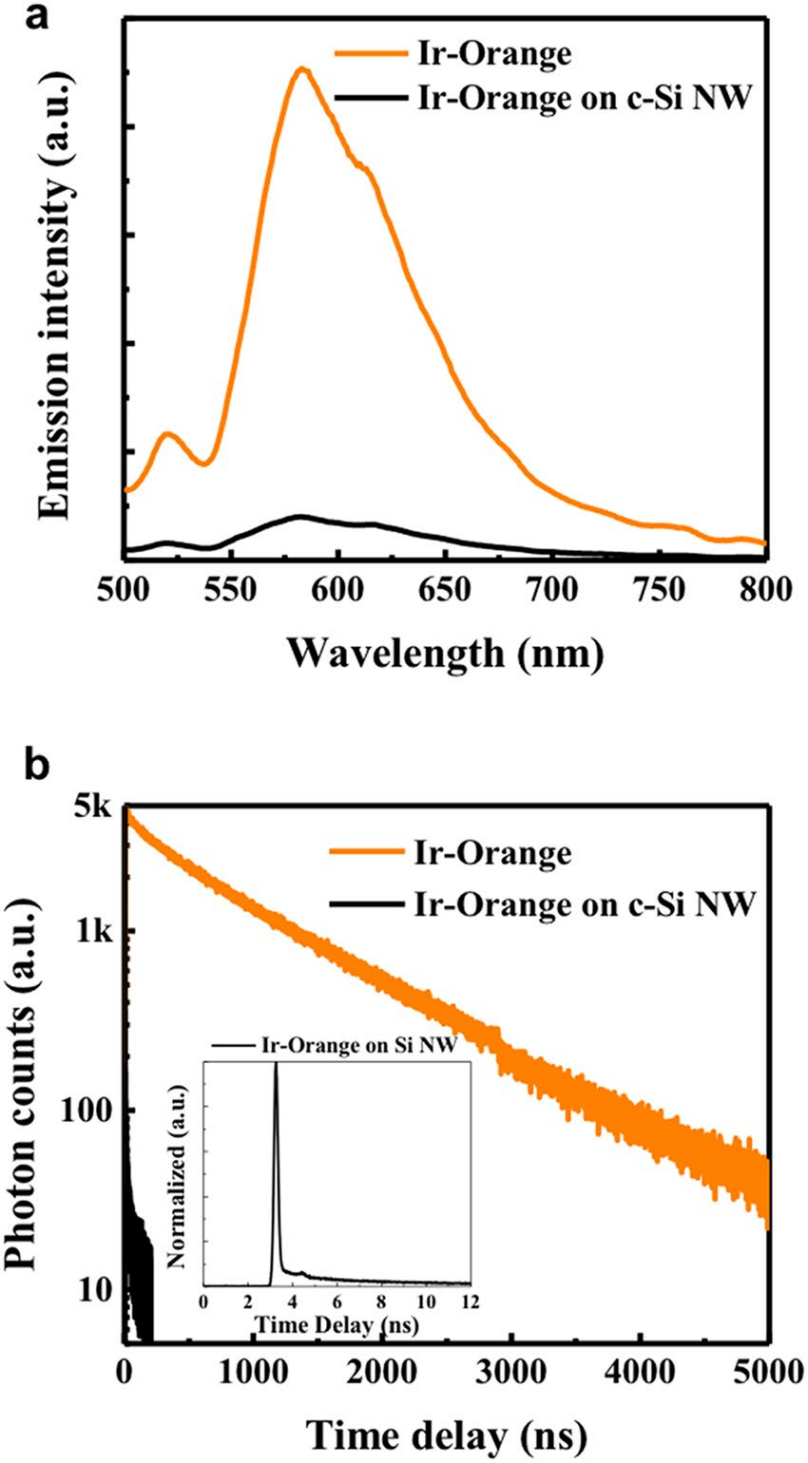
Analysis of energy downshifting effect by PL spectroscopy. (**a**) Steady-state PL spectra for Ir-Orange film on a quartz substrate (orange line) and on a c-Si NW substrate (black line). The intensity for the Ir-Orange film dramatically decreases when coated on a c-Si NW substrate. (**b**) Transient PL spectra for Ir-Orange film on a quartz substrate (orange line) and on a c-Si NW substrate (black line, and normalized intensity in inset). All films were coated by USD and were of the same thickness (20 nm).

To confirm the energy transfer from the Ir(III) complex-based energy downshifting layers to the c-Si NWSCs, both steady-state and transient photoluminescence (PL) spectra were analyzed via time-correlated single photon counting. The PL peaks of all the Ir(III) complex films on quartz substrates were strongly detected, whereas the emissions of all the films on the c-Si NWs were less pronounced when excited in the MLCT region, and the emission of the Ir-Orange film in particular almost disappeared (Fig. 2a and Supplementary Fig. 3). These results can be attributed to the long-range energy transfer from Ir(III) complex films to c-Si NWs through energy downshifting. This energy downshifting effect can be determined quantitatively by measuring the exciton lifetimes of the Ir(III) complexes with and without c-Si NWs via transient PL spectroscopy. The lifetimes of the pristine Ir(III) complexes were 612 ns for Ir-Red, 987 ns for Ir-Orange, 448 ns for Ir-Green, and 501 ns for Ir-Blue. The longer exciton lifetimes of the Ir(III) complexes compared with those of the quantum dots (~50 ns of II−VI QD, and ~7 ns of GQD) can enhance the energy downshifting efficiency^20,21,33,34^. On the other hand, when the Ir(III) complexes were coated on c-Si NWs, the exciton lifetimes of the Ir(III) complexes excited in the MLCT region dramatically decreased to 2 ns for Ir-Red, 6 ns for Ir-Orange, 2 ns for Ir-Green, and 3 ns for Ir-Blue (Fig. 2b, Supplementary Figs 4 and 5). Such a drastic decrease in the lifetime is direct evidence of long-range energy transfer. The long-range energy transfer efficiency can be calculated using (1 − T_AD_/T_D_) × 100%, where T_AD_ and T_D_ are the exciton lifetimes of the Ir(III) complexes with and without c-Si NWs, respectively^26^, and all the iridium-based energy downshifting layers studied here exhibited long-range energy transfer efficiencies of over 99%. These transient PL results are consistent with the emission decreases observed in the steady-state PL data.

### Device characterization

**Figure 3:**
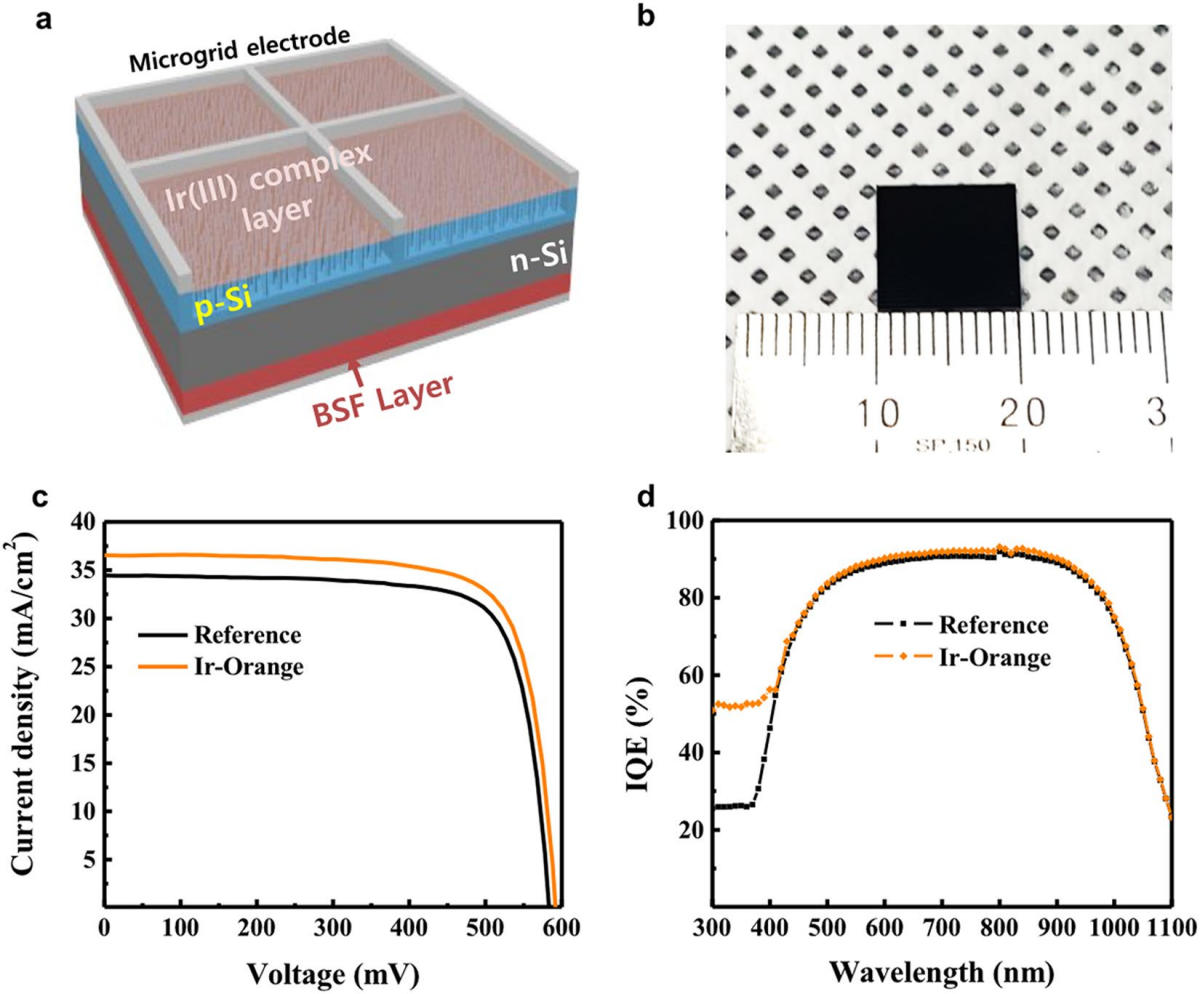
Device performance. (**a**) Structure of a c-Si NWSC fabricated with an Ir(III) complex-based energy downshifting layer. (**b**) Optical image of the c-Si NWSC. (**c**) *J–V* characteristics of devices employing Ir-Orange (orange line) and an uncoated reference device (black line). (**d**) IQE spectra of devices employing Ir-Orange (orange diamonds) and an uncoated reference device (black squares).

We then fabricated c-Si NWSCs adopting Ir(III) complex layers (Fig. 3a). First, to minimize the reflection at the front surface of c-Si solar cells, we applied nanowire arrays on the c-Si surface. The c-Si NW arrays were easily fabricated via the metal-assisted chemical etching (MACE) method^35^. Without additional treatments such as anti-reflection coating, the front surface of the solar cell looks almost black, as shown by the optical image in Fig. 3b, because the vertical NW arrays exhibit strong broadband optical absorption^36,37^. A p-type emitter layer was formed using a spin-on doping technique, and an n-type back surface field (BSF) layer was formed to minimize the near-infrared recombination on the rear side of the c-Si NWSCs. After the c-Si NWSCs were fabricated, Ir(III) complexes were coated on the surface of the c-Si NWSCs using a USD method. To evaluate the photovoltaic properties of the fabricated c-Si NWSCs, the current density–voltage (*J–V*) curves were measured under AM 1.5G illumination (Fig. 3c and Supplementary Fig. 6a), and the average photovoltaic parameters obtained from six replicate devices are listed in Table 1. As shown in Table 1, the c-Si NWSCs with Ir(III) complexes demonstrated a significantly enhanced short-circuit current density (*J*_*SC*_). This is because light with wavelengths longer than 500 nm is converted by the energy downshifting effect of the Ir(III) complex, as it passes through the SiNWs without any absorption loss and is then effectively absorbed by the c-Si bulk region where surface recombination is almost negligible^38,39^. Note that the c-Si NWSCs with Ir-Orange, which has the highest emission intensity, exhibited a maximum *J*_*SC*_ increase of 2.1 mA·cm^−2^ (6.10%) compared with uncoated reference devices. Therefore, our best device with Ir-Orange exhibited a PCE of 16.4% (*J*_*SC*_ = 36.5 mA·cm^−2^, open-circuit voltage (*V*_*OC*_) = 588 mV, and fill factor (FF) = 76.5%), which represents an increase of 5.81% compared with that of the reference device. In addition, the c-Si NWSCs coated with the other three Ir(III) complexes (i.e., Ir-Red, Ir-Green, and Ir-Blue) showed a *J*_*SC*_ increase of over 1.5 mA·cm^−2^ (Supplementary Fig. 6). The improvement in *J*_*SC*_ due to the energy downshifting effect can be more accurately elucidated from the internal quantum efficiency (IQE). The uncoated c-Si NWSCs exhibited a very low IQE of less than 40%, whereas the c-Si NWSCs coated with Ir-Orange exhibited drastically improved IQEs of over 55% in the short-wavelength range of 300–450 nm, as shown in Fig. 3d. Furthermore, the device performances of Ir(III) complex coated devices remained constant over a period of 8 days (Supplementary Fig. 8 and Supplementary Table 1). The enhanced performance can be ascribed to these reasons; i) the Ir(III) complexes efficiently absorb the UV light by MLCT, ii) the long exciton lifetime and high emission efficiency of the Ir(III) complex enhances the possibility of energy downshifting, and iii) the energy band gap of the Ir(III) complex includes that of silicon to facilitate long-range energy transfer from the Ir(III) complex into the silicon (Supplementary Scheme 1).

**Table 1:** Average photovoltaic parameters of c-Si NWSCs with USD-coated Ir(III) complexes (the values in brackets are obtained from the best device). The reference devices were fabricated without Ir(III) complexes.

Materials	*J*_*SC*_ (mA·cm^−2^)	*V*_*OC*_ (mV)	FF (%)	PCE (%)	Δ*J*_*SC*_ (%)	ΔPCE (%)
Reference	34.4 ± 0.1 (34.4)	588 ± 0.1 (588)	76.3 ± 0.3 (76.6)	15.4 ± 0.1 (15.5)	—	—
Ir-Red	36.2 ± 0.1 (36.2)	588 ± 0.1 (588)	76.0 ± 0.5 (76.5)	16.2 ± 0.1 (16.3)	5.23 (5.23)	5.19 (5.16)
Ir-Orange	36.4 ± 0.1 (36.5)	588 ± 0.1 (588)	76.5 ± 0.3 (76.5)	16.4 ± 0.1 (16.4)	5.81 (6.10)	6.49 (5.81)
Ir-Green	35.8 ± 0.1 (35.9)	588 ± 0.1 (588)	76.4 ± 0.1 (76.5)	16.1 ± 0.1 (16.2)	4.07 (4.36)	4.55 (4.52)
Ir-Blue	35.9 ± 0.1 (36.0)	588 ± 0.1 (588)	76.4 ± 0.2 (76.5)	16.1 ± 0.1 (16.2)	4.36 (4.65)	4.55 (4.52)

^a^The parameters given are averages obtained from six replicate devices for each material.

To clarify the energy downshifting effect in c-Si NWSC, we introduced a reference, TIr2, which has almost no effect on the energy downshifting system^40^. As a result, *J*_*sc*_ and PCE performances of a TIr2 coated device were almost the same as that of a non-coated control device in its J-V curve and EQE data (Supplementary Fig. 9). Furthermore, the total reflectance of c-Si NWs with or without the Ir(III) complex layer was not different, thereby there is no anti-reflection effect (Supplementary Fig. 10).

### Morphology study of energy downshifting layer by USD

**Figure 4:**
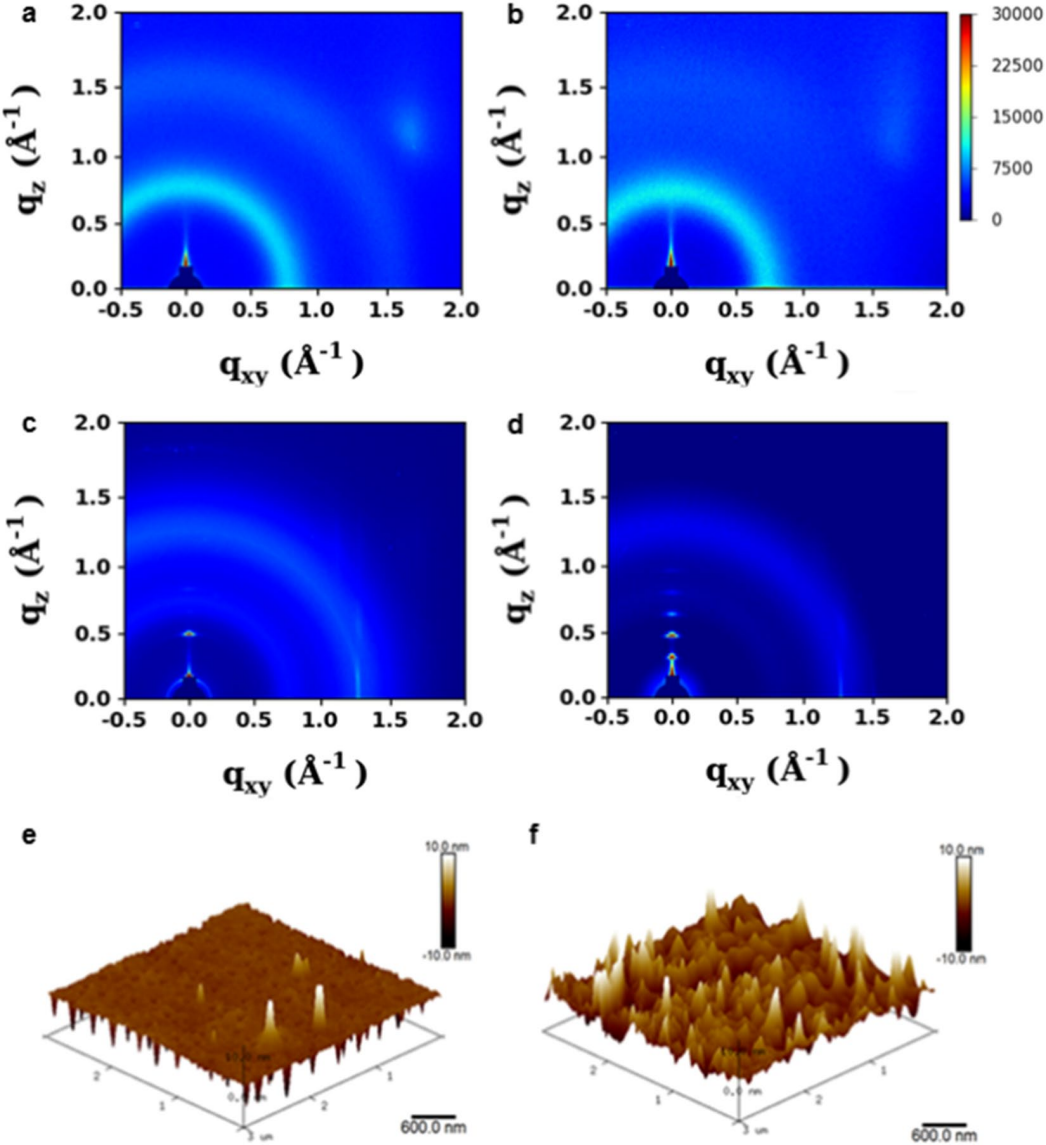
GIWAXD patterns of Ir(III) complex downshifting layers. 2D GIWAXD diffraction patterns of (**a**) Ir-Orange and (**b**) Ir-Blue films by conventional spin coating, and (**c**) Ir-Orange and (**d**) Ir-Blue films by ultrasonic spray deposition. 3D AFM images of (**e**) USD-coated Ir-Orange and (**f**) spin-coated Ir-Orange films on c-Si NW substrates.

The synchrotron X-ray diffraction results clearly revealed an ordered aggregation of these Ir(III) complexes in thin films deposited by USD. Grazing-incidence wide-angle X-ray diffraction (GIWAXD) was performed to investigate the crystal structure, orientation, and molecular packing of Ir(III) complex-based energy downshifting layers. Previous reports on organic light emitting diodes indicate that photoluminescence efficiency is affected by the crystallinity of phosphor because crystalline phosphors exhibit much lower non-radiative recombination than amorphous phosphors^41–43^. Figure 4a–d show the two-dimensional (2D) GIWAXD patterns of Ir-Orange and Ir-Blue films. The Ir(III) complex films formed by a conventional spin-coating method exhibited amorphous structures (Fig. 4a,b and Supplementary Figs 11a–d), whereas the films formed by USD exhibited highly crystalline structures with a strong diffraction spots along the out-of-plane (*q*_*z*_) direction; this is because the USD method achieves a continuous uniform deposition by spraying atomized droplets (diameter ~8 μm, see the calculation section in the Supporting Information). Thus, highly crystalline Ir(III) complex thin films formed by USD exhibited much more intense PL than the films formed by spin coating because the high crystallinity of Ir(III) complexes contributes to defect passivation and increases the radiative decay (Supplementary Fig. 12)^41,43^. Therefore, the enhancement in *J*_*SC*_ was lower for devices employing spin-coated Ir(III) complex films compared with that for devices employing Ir(III) complex films coated by USD (Supplementary Fig. 13 and Supplementary Table 2). Interestingly, the amorphous Ir-Blue film formed by spin coating exhibited a very strong PL intensity because the difluorinated ligand of Ir-Blue has a perfluorination effect that decreases the number of high-frequency vibrations associated with C–H stretching, thereby enhancing the radiative decay^41,44^. Therefore, devices with Ir-Blue demonstrated the best performance among spin-coated devices (*J*_*SC*_ = 35.7 mA·cm^−2^, PCE = 15.2%).

To investigate the scattering effect of the Ir(III) complex layers, we measured the reflectance and transmission of the Ir(III) complex layers formed by either spin coating or USD coating. As a result, there was no difference in reflectance or transmittance of the Ir(III) complex layers, which nearly reached 100% overall across the wavelength region (Supplementary Fig. 14). Therefore, there is almost no scattering effect in both of the USD coated and spin coated samples.

From 3D atomic force microscopy (AFM) and scanning electron microscopy (SEM) analyses, it is confirmed again that the Ir-Orange film formed on c-Si NWs by USD has a much more uniform and fine surface morphology than the spin-coated Ir-Orange film, which leads to a lower root-mean-square value for USD-coated Ir-Orange films (9.24 nm) than that for spin-coated Ir-Orange films (24.3 nm) (Fig. 4e,f and Supplementary Fig. 15). Furthermore, the USD-coated Ir-Orange thin film penetrated very well into the vertical c-Si NW arrays, as shown in Fig. 4e, which would significantly increase the active surface area and thus further enhance the energy downshifting effect.

## Discussion

In conclusion, we designed phosphorescent energy downshifting systems based on four different Ir(III) complexes to diminish surface recombination in c-Si NWSCs. The Ir(III) complex-based thin films were employed as energy downshifting layers in c-Si NWSCs, which can convert UV light to visible light. Transient PL spectroscopy results showed that all the Ir(III) complexes employed in c-Si NWSCs exhibited long-range energy-transfer efficiencies of over 99%. The effective energy transfer via the Ir(III) complex layer improved the *J*_*SC*_ value (from 34.4 to 36.5 mA·cm^−2^), and a dramatic increase in IQE from ~40% to ~55% was obtained in the short-wavelength region of 350–450 nm, resulting in a significantly improved PCE (16.4%) compared with that of a reference device without the Ir(III) complex layer (15.5%). Moreover, according to the GIWAXD study, highly crystalline thin films of Ir(III) complexes formed by USD exhibited higher PL intensities than films prepared by a conventional spin-coating method, which resulted in a greater increase in the *J*_*SC*_ value of devices employing Ir(III) complex films coated by USD. We believe that various molecular engineering strategies of Ir(III) complexes would open up many opportunities for the design and development of energy downshifting systems to overcome the limitations of previous photon utilization system in various optoelectronic applications.

## Methods

### Synthesis of Ir(III) complexes

A solution of IrCl_3_·nH_2_O and 1-phenylisoquinoline (1pq for Ir-Red) ligand in 2-methoxyethanol/H_2_O (3:1, v/v%) was refluxed for 24 hours under an inert condition of nitrogen. After the solution was cooled down to room temperature, more water was added to precipitate the product. The precipitation was filtered out through Büchner funnel. That solid was washed with three times n-hexane and cold diethyl ether to get the product, (1pq)_4_Ir_2_Cl_2_ dimer (Scheme S2). A mixture of (1pq)_4_Ir_2_Cl_2_ (500 mg, 0.40 mmol) and 3-hydroxypicolinic (168 mg, 1.21 mmol) acid and Na_2_CO_3_ (423 mg, 4.00 mmol) was refluxed under inert condition of nitrogen for 12 hours. After the solution was cooled down to room temperature, solution was evaporated under reduced pressure. The solid was dissolved in methylene chloride and then it washed with water and dried over by MgSO_4_. The solvent was evaporated to get crude product and it was purified by column chromatography on silica gel. At a result, the product (Ir-Red) was provided (422.4 mg, 71.3% yield). Other Ir(III) complexes were synthesized with same procedure with Ir-Red in the above (Supplementary Scheme 2).

### Steady-state optical study

Dichloromethane was used to prepare 0.02 mM solutions of each Ir(III) complex solutions. A 1 cm * 1 cm cell (Hellma) and 1 cm * 0.2 cm cell (Hellma) were used for absorbance and emission analysis of solution state. A 2 cm * 2 cm quartz substrates were used for the optical analysis of Ir(III) complex films. An UV–visible spectrophotometer (Agilent Cary 100), fluorescence spectrophotometer (Varian Cary Eclipse), and integrated sphere spectrofluorometer (Jasco International) were used to measure the absorbance, emission, and quantum yield, respectively. In the analysis of emission, the excited wavelength was in the MLCT region of each Ir(III) complexes, 376 (Ir-Blue), 400 (Ir-Green), 455 (Ir-Orange), and 462 nm (Ir-Red) (Supplementary Fig. 16).

### Transient PL measurement

Transient PL data were obtained via time-correlated single photon counting (TCSPC) (Supplementary Fig. 17). Excitation of the sample was achieved using ~150 fs pulses provided by second harmonic generation (SHG = 360 nm and 420 nm) from a tunable Ti:sapphire laser, Mira900 (Coherent Inc., CA, USA) at a 76-MHz repetition rate. Emitted photons of various wavelengths generated by radiative decay were resolved using a monochromator, Acton series SP-2150i (Princeton Instruments, MA, USA). The total instrument response function for photon decay was less than 150 ps, and the lifetime of the phosphorescence decay of the Ir(III) complexes was fitted using the FluoFit software, assuming that the χ^2^ value is approached to 1 but less than 1 if it is reasonable.

### Fabrication of Si nanowires via metal-assisted chemical etching

Si nanowires were fabricated on the front side of n-type Czochralski Si wafers (resistivity: 1–3 Ω cm, thickness: 400 μm) by metal-assisted chemical etching. The Si substrate was dipped into a diluted hydrofluoric acid (HF) solution to remove the native oxide on the substrate. Ag nanoparticles were then deposited onto the hydrogen-terminated Si substrate by electroless deposition in a mixed solution containing 4.8 M HF and 0.005 M silver nitrate (AgNO_3_) for 5 s at room temperature. After the deposition process, the substrate was immersed in an aqueous solution containing 4.8 M HF and 0.6 M hydrogen peroxide (H_2_O_2_) for 20 s at room temperature. Si nanowires were then obtained by dipping the substrate in diluted nitric acid (HNO_3_) for 10 min to remove the Ag nanoparticles.

### Fabrication of Si nanowire solar cells

The emitter and back surface field (BSF) layers were formed by a spin-on dopant (SOD) method. First, a phosphorus dopant source (P509, Filmtronics, Inc.) for the BSF layer was spin coated onto a dummy Si wafer, which was then baked on a hotplate at 200 °C for 20 min. To form a uniform BSF layer on the back side of the Si wafer, we positioned the Si wafer so that it faced the phosphorus-coated dummy wafer. The diffusion doping of phosphorus was then performed in a tube furnace under a mixed ambient atmosphere of 20% O_2_ and 80% N_2_ at 900 °C. The phosphorus silicate glass, which remained even after the SOD diffusion, was removed by dipping the Si wafer in a diluted HF solution. Subsequently, the emitter layer was formed on the front side of the Si wafer using a boron dopant source (B155, Filmtronics, Inc.). The boron dopant source was spin coated onto a dummy Si wafer, which was then baked on a hotplate at 200 °C for 20 min. For conformal doping in the nanowire structure, we positioned the Si wafer so that it faced the boron-coated dummy wafer. Boron doping was then conducted in a tube furnace under a N_2_ atmosphere at 880 °C. The boron silicate glass and native silicon oxide were removed by dipping the Si wafer in a diluted HF solution. The Si structures were covered with a photoresist (AZ4330, AZ electronic materials) before Al deposition to form the front metal electrode. The microgrid (a microscale mesh structure) was patterned using a photolithography process. For the top and bottom contacts, 500 nm-thick Al films were deposited onto the top and bottom of the substrate using a thermal evaporator. Finally, the photoresist was removed by dipping the Si wafer in an acetone solution.

### Characterization of Si nanowire solar cells

The surface morphologies of the Si structures were characterized using field-emission scanning electron microscopy (FE-SEM, Hitachi S-4800). The photovoltaic properties of the solar cells were investigated using a solar simulator (Class AAA, Oriel Sol3A, Newport) under AM 1.5 G illumination. The incident flux was measured using a calibrated power meter and double-checked using a solar cell calibrated by the National Renewable Energy Laboratory (PV Measurements, Inc.). EQE measurements were performed using a Xe light source and a monochromator in a wavelength range of 300–1100 nm. Optical reflection measurements were conducted over wavelengths of 300–1100 nm using a UV–vis/NIR spectrophotometer (Cary 5000, Agilent) equipped with a 110-mm integrating sphere to account for the total light (diffuse and specular) reflected by the samples.

### Ultrasonic spray deposition (USD)

The concurrently pumped ultrasonic spray coating was performed using an ExactaCoat system fixed with a pair of 180 kHz impact ultrasonic nozzles (Sono-Tek Corp.). The prepared precursor solutions in chloroform solvent (SAMCHUN Chemicals, Korea) were fed into the ultrasonic nozzle at a spray rate of 0.1 mL min^−1^. A nozzle-to-substrate distance of 10 cm and a compressed N_2_ gas pressure of 3.0 psi were used to spray the c-Si NW surface or a quartz substrate at a spray rate of 20 mm s^−1^ on a temperature-controlled (100 °C) stage.

### Morphological study

In SEM, AFM, and GIWAXD measurements, the thicknesses of active layers under all conditions were identical (15 nm). The surface morphology of Ir(III) complex films was investigated using SEM (S-2700, Hitachi, Japan). AFM images were obtained using a Veeco microscope in tapping mode over a scan area of 1 mm × 1 mm. 2D GIWAXD measurements were performed at the PLS-II 6D UNIST-PAL beamline of the Pohang Accelerator Laboratory in Korea. The X-ray coming from the bending magnet was monochromated (*λ* = 1.06 Å) using a double-crystal monochromator (DCM) and focused using a sagittal focusing DCM and bendable toroidal mirror system (beam size: 150 μm (H) × 120 μm (V) in FWHM @ sample position). The 2D GIWAXD measurement system was equipped with a six-axis motorized sample stage inside a vacuum chamber (~2 × 10^−2^ Torr) to enable the fine alignment of a thin-film sample and effective removal of unwanted air and window scattering. The 2D GIWAXD patterns were recorded using a 2D CCD detector (Rayonix MX225-HS, USA), and the diffraction angles were calibrated using a pre-calibrated sucrose sample (monoclinic, P21, a = 10.8631 Å, b = 8.7044 Å, c = 7.7624 Å, and *β* = 102.938°).

## Electronic supplementary material

Supplementary information

## Data Availability

The data supporting the findings of this study are available within the article and its Supplementary Information files. All other relevant source data are available from the corresponding author upon reasonable request.
