# Supplementary material for: Phosphorescent Energy Downshifting for Diminishing Surface Recombination in Silicon Nanowire Solar Cells
Source: Sci Rep. 2018 Nov 19;8:16974. doi: 10.1038/s41598-018-35356-w (PMC6242905; doi:10.1038/s41598-018-35356-w)
Supplement: Supplementary file 1 — Supplementary information [file 41598_2018_35356_MOESM1_ESM.docx]

Phosphorescent Energy Downshifting System for Diminishing Surface Recombination in Silicon Nanowire Solar Cells

Hyun-Tak Kim,^†,ǁ^ Kangmin Lee,^‡,ǁ^ Wonjoo Jin,^‡^ Han-Don Um,^‡^ Minsoo Lee,^†^ Eunhye Hwang,^†^ Tae-Hyuk Kwon^*,†^, and Kwanyong Seo,^*,‡^

†Department of Chemistry, Ulsan National Institute of Science and Technology, Ulsan, Republic of Korea

‡Department of Energy Engineering, Ulsan National Institute of Science and Technology, Ulsan, Republic of Korea

*e-mail: [kwon90@unist.ac.kr](mailto:kwon90@unist.ac.kr), [kseo@unist.ac.kr](mailto:kseo@unist.ac.kr)

**Contents**

1. Energy downshifting process in c-Si NWSC (2)
2. Synthesis of Ir(III) complexes (3)
3. Steady state PL spectra of Ir(III) complexes (5)
4. Energy diagrams of Ir(III) complexes and Si (6)
5. Steady state PL spectra of Ir(III) complexes on c-Si nanowire (7)
6. Transient PL spectra of Ir(III) complexes on c-Si nanowire (8)
7. J-V curves and IQE data of devices employing Ir(III) complexes (10)
8. EQE spectra of devices employing Ir(III) complexes (11)
9. Light stability of devices employing Ir(III) complexes (12)
10. Performance of TIr2 coated device and characterization of TIr2 (13)
11. Total reflectance of Ir(III) complexes on c-Si NWSCs (14)
12. GIWAXD analysis for films of Ir(III) complexes by (15)
13. Micro droplet size of USD calculation (16)
14. PL enhanced effect by USD (17)
15. Device performances (18)
16. Reflectance and transmittance spectra of devices with Ir(III) complexes layers (19)
17. SEM images of Ir-Orange films on c-Si nanowire (20)
18. Description of PL measurement (21)
19. Table (23)
20. References (25)

**Mechanism of Ir(III) complex based energy downshifting system in c-Si NWSC**


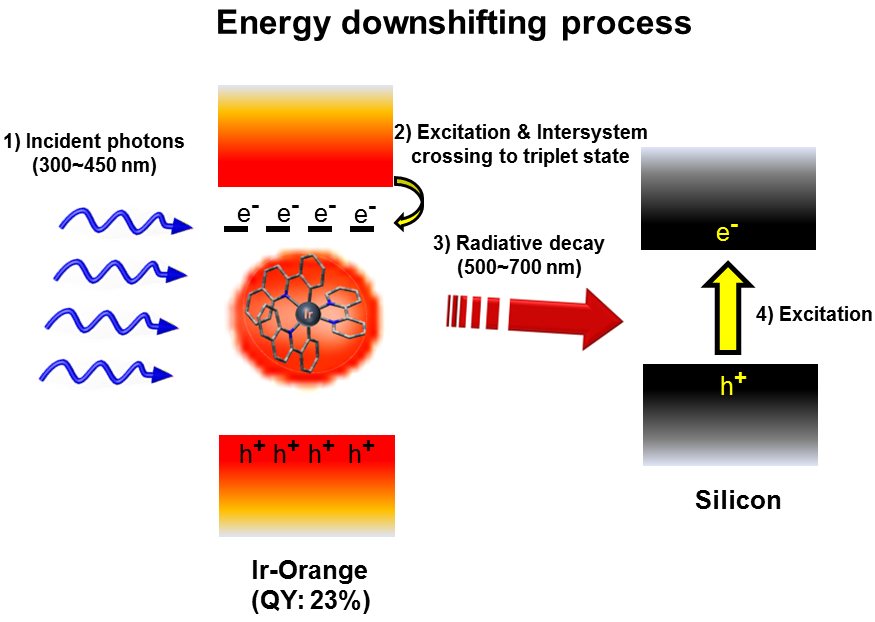


**Supplementary Scheme 1. Ir(III) complex based energy downshifting process in c-Si NWSC.**

**Synthesis and characterization of Ir-Red, Ir-Orange, Ir-Green, and Ir-Blue complexes**


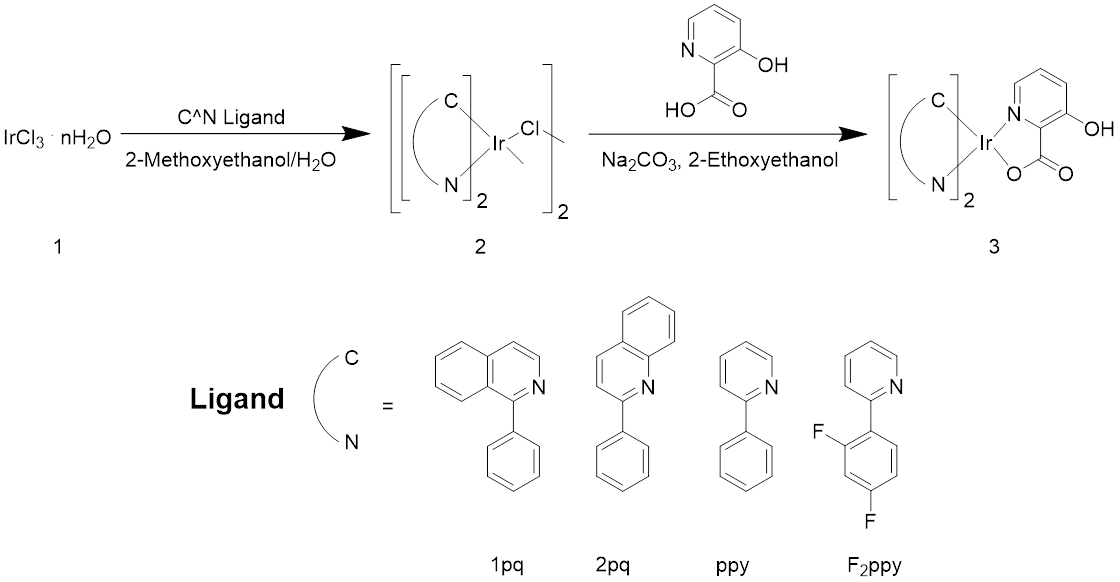


**Supplementary Scheme 2. Synthetic pathway of four Ir(III) complexes.**

**Synthesis of [(C^N)_4_Ir_2_Cl_2_].** A solution of IrCl_3_·nH_2_O and C^N ligand in 2-methoxyethanol/H_2_O (3:1, v/v%) was refluxed for 24 hours under an inert condition of nitrogen. After the solution was cooled down to room temperature, more water was added to precipitate the product. The precipitation was filtered out through Büchner funnel. That solid was washed with three times n-hexane and cold diethyl ether to get the product. Those dimers (compound **2**) were synthesized by the previously reported method of Nonoyama.^1^

**Synthesis of Ir-Red, Ir-Orange, Ir-Green, and Ir-Blue.** A mixture of (C^N, 1pq)_4_Ir_2_Cl_2_ (500 mg, 0.40 mmol) and 3-hydroxypicolinic (168 mg, 1.21 mmol) acid and Na_2_CO_3_ (423 mg, 4.00 mmol) was refluxed under inert condition of nitrogen for 12 hours. After the solution was cooled down to room temperature, solution was evaporated under reduced pressure. The solid was dissolved in methylene chloride and then it washed with water and dried over by MgSO_4_. The solvent was evaporated to get crude product and it was purified by column chromatography on silica gel. As a result, the product (Ir-Red) was provided (422.4 mg, 71.3% yield).^2^

Other Ir(III) complexes ((2pq)_2_Irpic-OH (Ir-Orange), (ppy)_2_Irpic-OH (Ir-Green), (F_2_ppy)_2_Irpic-OH (Ir-Blue)) were synthesized with similar procedure with Ir-Red in the above.

**Ir-Red: (1pq)_2_Irpic-OH**

^1^H NMR (400 MHz, CDCl_3_): δ 13.84 (s, 1H), 8.96 (m, 2H), 8.66 (d, *J*=6.4 Hz, 1H), 8.26 (d, *J*=7.6 Hz, 1H), 8.19 (d, *J*=7.6 Hz, 1H), 7.94 (m, 1H), 7.880 (m, 1H), 7.73 (m, 4H), 7.52 (d, J=6.8 Hz, 1H), 7.45 ( d, J=6.4 Hz, 1H), 7.367 (m, 1H), 7.32 (d, *J*=6.4 Hz, 1H), 7.16 (m, 1H), 7.10 (m, 1H), 7.01 (m, 1H), 6.94 (m, 1H), 6.77 (m, 1H), 6.72 (m, 1H), 6.49 (d, *J*=6.8 Hz, 1H), 6.22 (d, *J*=6.8 Hz, 1H).

**Ir-Orange: (2pq)_2_Irpic-OH**

^1^H NMR (400 MHz, CDCl_3_): δ 13.42 (s, 1H), 8.65 (d, *J*=8.8 Hz, 1H), 8.21 (d, *J*=16.8 Hz, 1H), 8.19 (d, *J*=16.8 Hz, 1H), 8.11 (m, 2H), 7.95 (dd, *J*=8 Hz, 1H), 7.84 (dd, *J*=8 Hz, 1H), 7.74 (m, 2H), 7.55 (m, 1H), 7.47 (m, 2H), 7.36 (m, 2H), 7.12 (m, 2H), 7.10 (m, 1H), 6.99 (m, 1H), 6.95 (m, 1H), 6.86 (m, 1H), 6.77 (m, 1H), 6.666 (m, 1H), 6.29 (dd, *J*=6.8 Hz, 1H).

**Ir-Green: (ppy)_2_Irpic-OH**

^1^H NMR (400 MHz, CDCl_3_): δ 13.81. (s,1H), 8.72 (m, 1H), 7.89 (d, *J*=17.2 Hz, 1H), 7.87 (d, *J*=16.8 Hz, 1H), 7.75 (m, 2H), 7.61 (m, 2H), 7.52 (m, 1H), 7.38 (m,1 H), 7.25 (m, 1H), 7.18 (m, 2H), 6.99 (m, 1H), 6.94 (m, 1H), 6.89 (m, 1H), 6.81 (m, 1H), 6.77 (m, 1H), 6.37 (dd, *J*=7.6 Hz, 1H), 6.20 (dd, *J*=7.6 Hz, 1H).

**Ir-Blue: (F_2_ppy)Irpic-OH**

^1^H NMR (400 MHz, CDCl_3_): δ 13.57 (s, 1H), 8.67 (m, 1H), 8.31 (m, 1H), 8.26 (d, *J*=8.4 Hz, 1H), 7.82 (m, 2H), 7.48 (m, 1H), 7.45 (m, 1H), 7.26 (m, 2H), 7.24 (m, 1H), 7.05 (m, 1H), 6.51 (m, 1H), 6.44 (m, 1H), 5.79 (m, 1H), 5.59 (m, 1H).

The ^1^H NMR results were well matched with that of our previous report for synthesizing the Ir(III) complexes.^3^

**Steady state PL of Ir(III) complexes films on quartz substrate and solutions in CH_2_Cl_2_.**

**
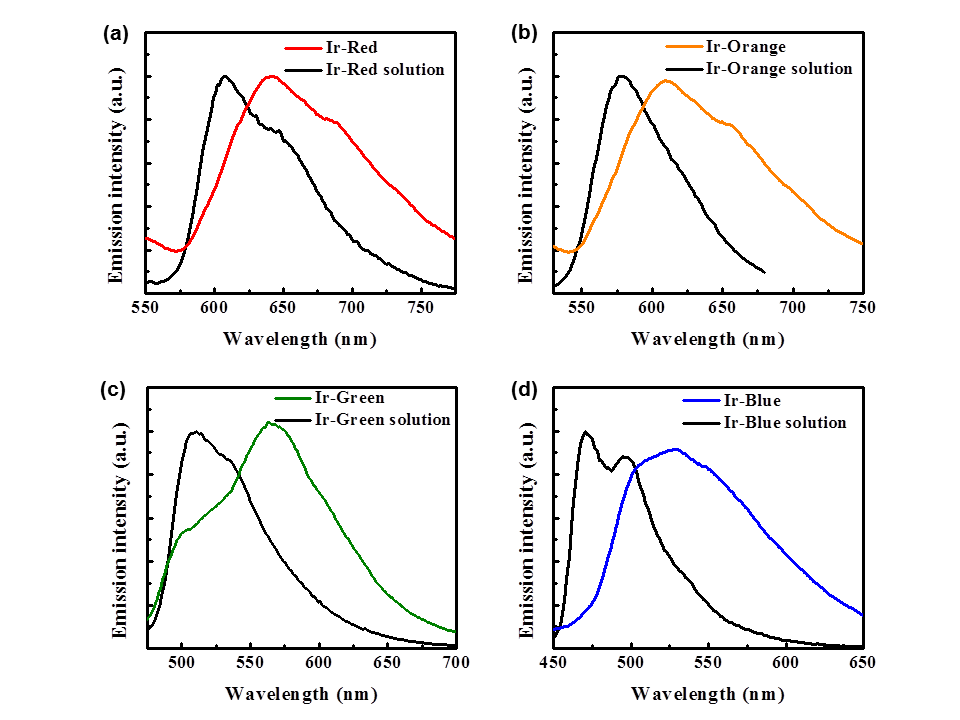
 Supplementary Figure 1. Emission intensities of Ir(III) complexes film on quartz by USD and Ir(III) complexes 20 μM solution in CH_2_Cl_2_. a-d.** Black lines are steady state PL spectra of (a) Ir-Red, (b) Ir-Orange, (c) Ir-Green, and (d) Ir-Blue 20 μM solution in CH_2_Cl_2_ and red, orange, green, and blue lines are steady state PL spectra of (a) Ir-Red, (b) Ir-Orange, (c) Ir-Green, and (d) Ir-Blue films on quartz by USD, respectively. (When excited at their MLCT region under the same slit width condition (excitation: 10 nm and emission: 10 nm)).

**Energy diagrams of Ir(III) complexes and p-type Si.**


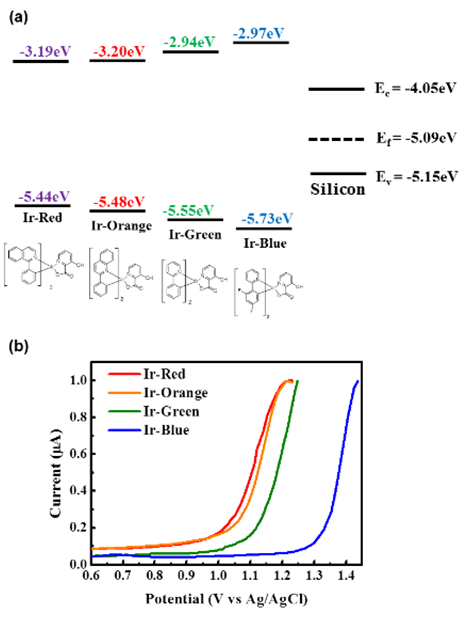


**Supplementary Figure 2. Energy diagrams of Ir-Red, Ir-Orange, Ir-Green, Ir-Blue, and p-type Si. a, b.** (a) The energy band gap of the Ir(III) complexes is wider than that of the p-type Si. Thus, the absorbed light energy can be transferred from Ir(III) complexes to p-type Si. Highest occupied molecular orbital (HOMO) and lowest unoccupied orbital (LUMO) of Ir(III) complexes were calculated by (b) cyclic voltammetry and UV-visible absorption spectroscopy (Fig. 1c).

**Steady state PL of Ir(III) complexes on quartz substrate and on c-Si nanowire**


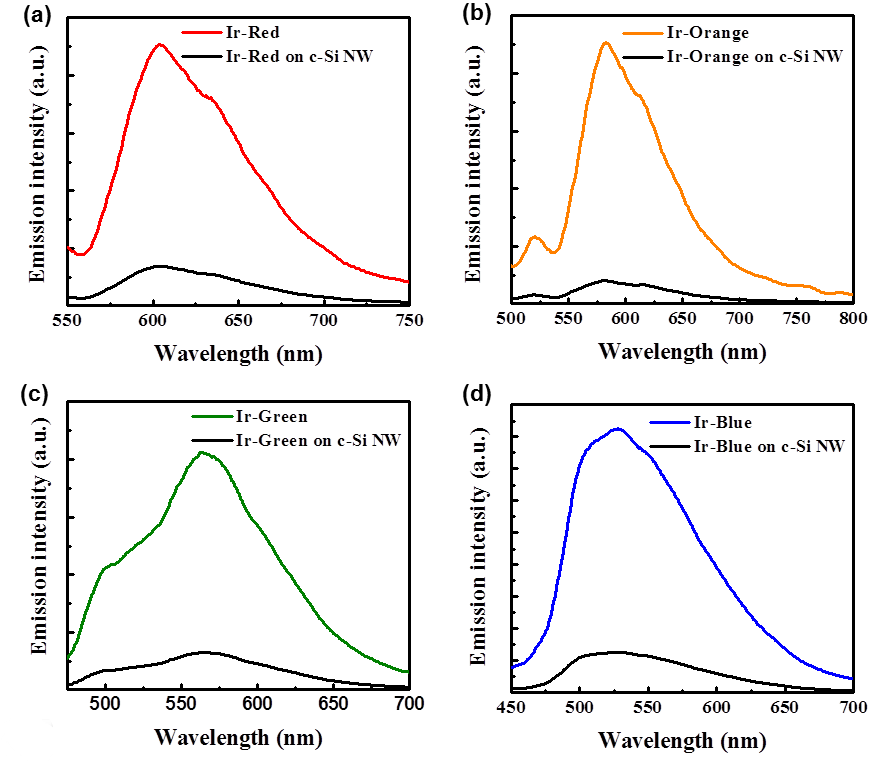


**Supplementary Figure 3. Emission intensities of Ir(III) complexes film on quartz and on c-Si NW by USD, a-d.** Black lines are steady state PL spectra of (a) Ir-Red, (b) Ir-Orange, (c) Ir-Green, and (d) Ir-Blue on c-Si NW and red, orange, green, and blue lines are steady state PL spectra of (a) Ir-Red, (b) Ir-Orange, (c) Ir-Green, and (d) Ir-Blue on quartz, respectively.

**Transient PL of Ir(III) complexes on quartz substrate and on c-Si nanowire**


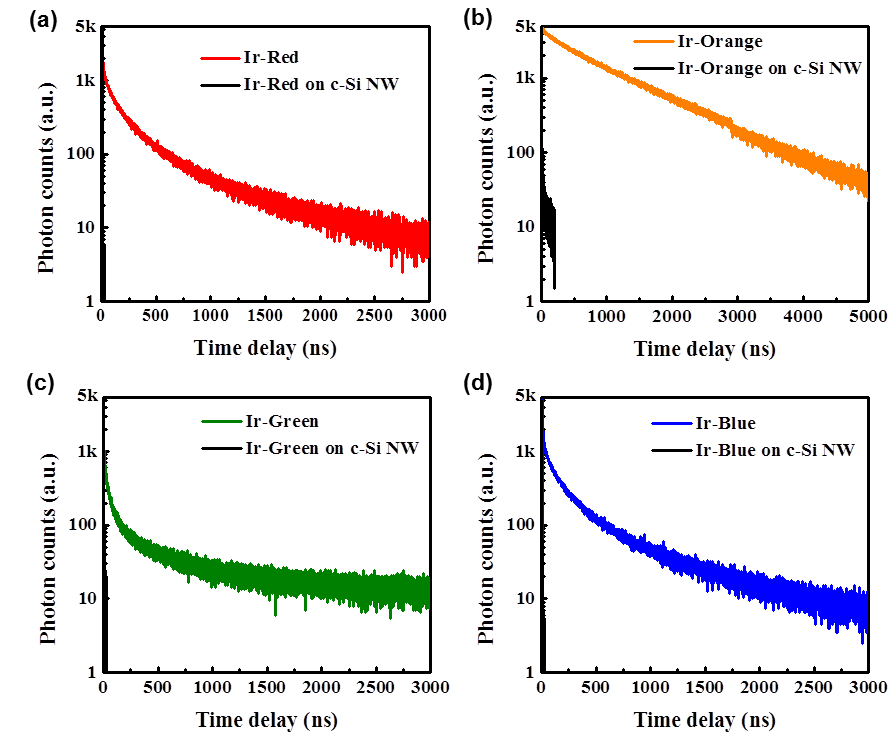


**Supplementary Figure 4. Exciton lifetime of Ir(III) complexes film on quartz and on c-Si NW by USD, a-d.** Black lines are transient PL spectra of (a) Ir-Red, (b) Ir-Orange, (c) Ir-Green, and (d) Ir-Blue on c-Si NW and red, orange, green, and blue lines are transient PL spectra of (a) Ir-Red, (b) Ir-Orange, (c) Ir-Green, and (d) Ir-Blue on quartz, respectively.

**Transient PL of Ir(III) complexes on quartz substrate and on c-Si nanowire**


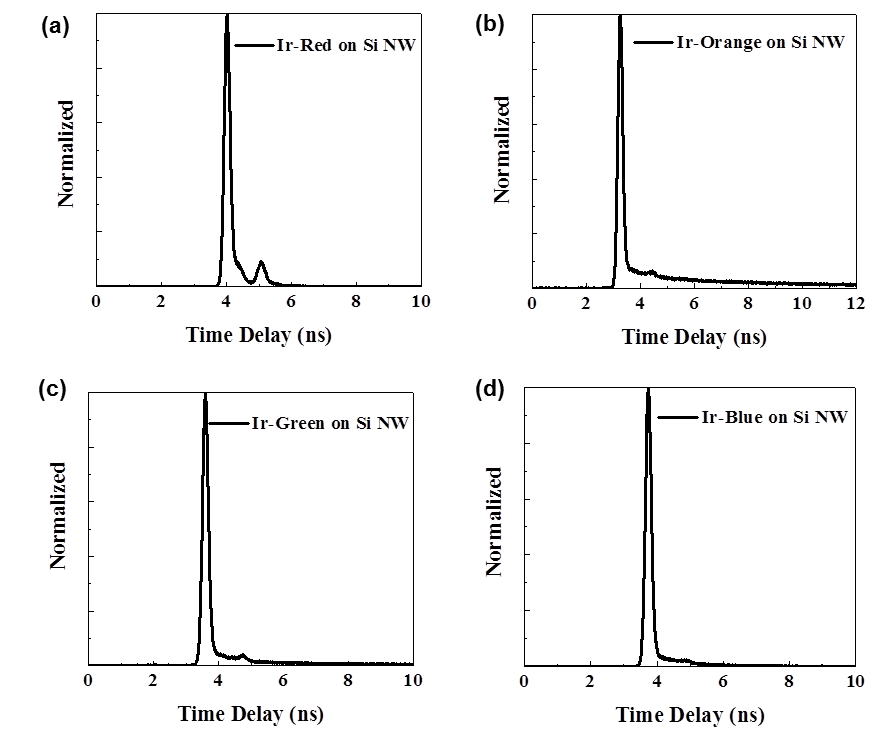


**Supplementary Figure 5. Exciton lifetime of Ir(III) complexes film on c-Si NW by USD, a-d.** Transient PL spectra of (a) Ir-Red, (b) Ir-Orange, (c) Ir-Green, and (d) Ir-Blue on c-Si NWSC.

**J-V curves and IQE data of devices employing Ir(III) complexes**


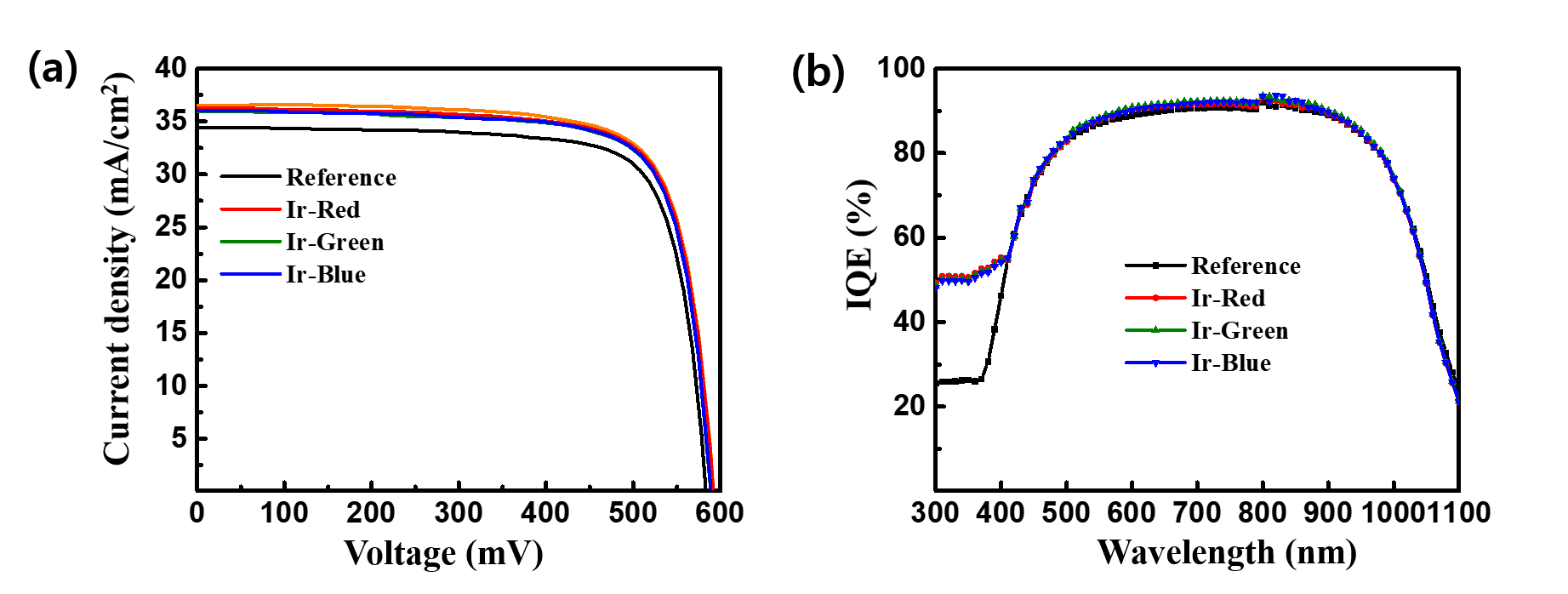


**Supplementary Figure 6. Device characterization.** (a) *J–V* characteristics of devices employing Ir-Red (red line), Ir-Green (green line), and Ir-Blue (blue line) and an uncoated reference device (black line). (b) IQE spectra of devices employing Ir-Red (red circles), Ir-Green (green upward triangles), and Ir-Blue (blue downward triangles) and an uncoated reference device (black squares).

**External quantum efficiency (EQE) spectra of c-Si NWSCs with and without Ir(III) complexes**


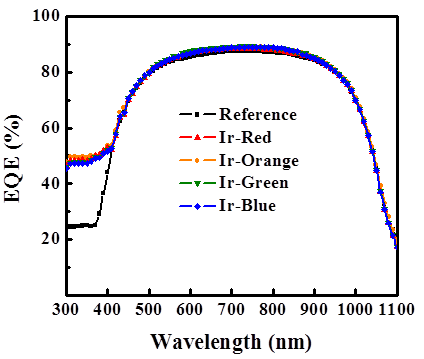


**Supplementary Figure 7.** **EQE spectra of devices employing Ir-Red (red upward triangles), Ir-Orange (orange circles), Ir-Green (green downward triangles), and Ir-Blue (blue diamonds) and an uncoated reference device (black squares).**

**Light stability**


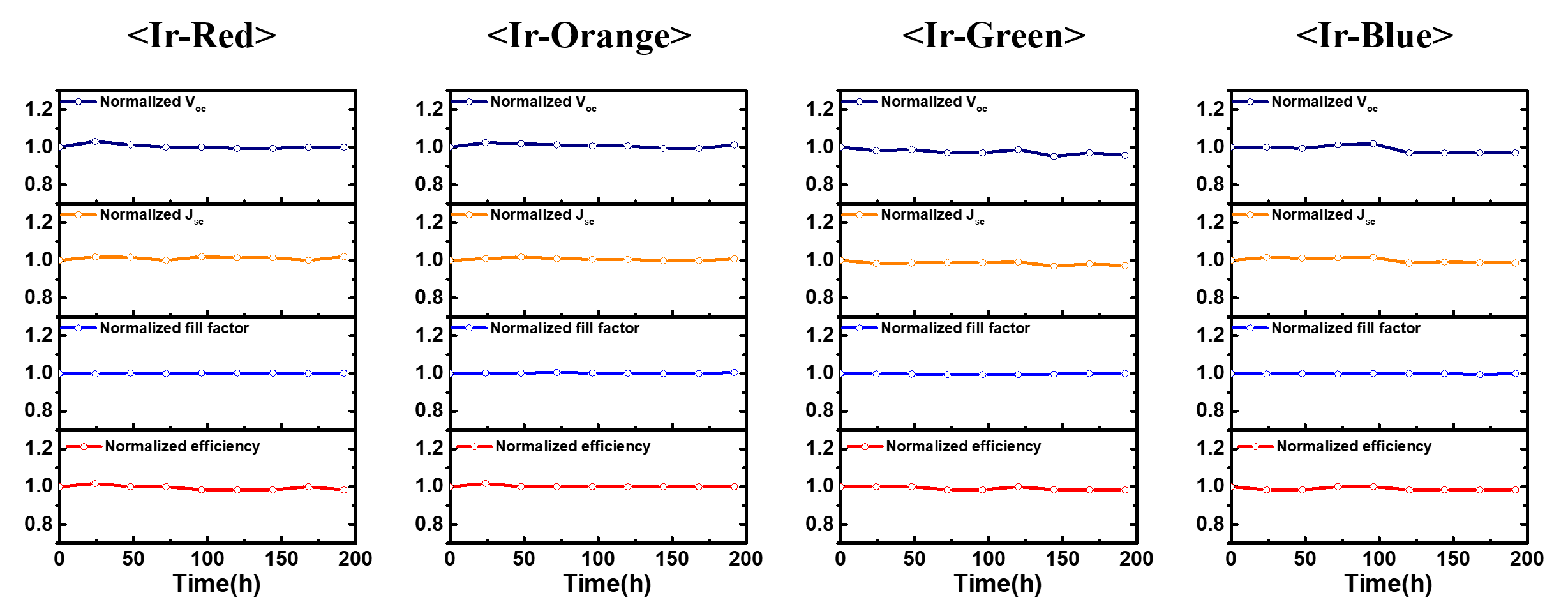
 **Supplementary Figure 8. Light stability of the devices coated with Ir-Red, Ir-Orange, Ir-Green, and Ir-Blue over an 8 day period.**

**Performance of TIr2 coated device and characterization of TIr2**


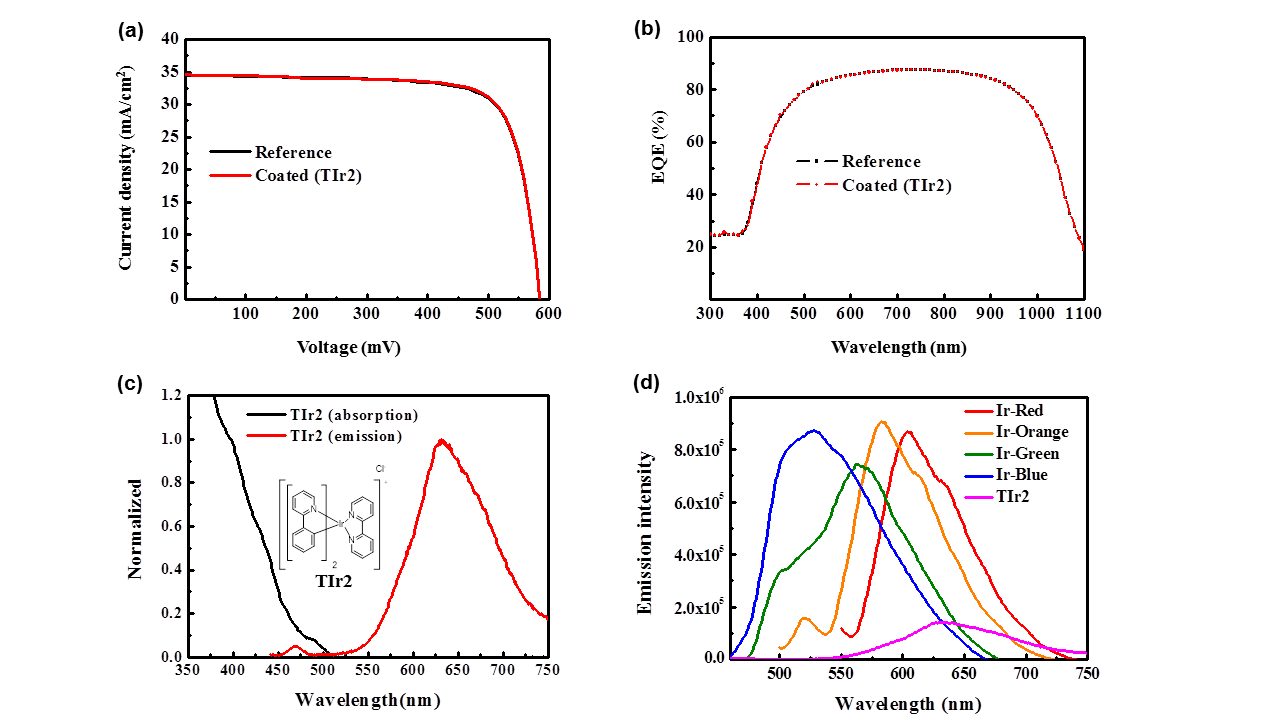


**Supplementary Figure 9. Device performance of TIr2**. (a) *J–V* characteristics of devices employing TIr2 (red line) and an uncoated reference device (black line). (b) EQE spectra of devices employing TIr2 (red circle) and an uncoated reference device (black squares). (c) Absorption (black line) and PL (red line) spectra of USD-coated TIr2 film. (d) PL spectra of Ir-Red (red line), Ir-Orange (orange line), Ir-Green (green line), Ir-Blue (blue line), and TIr2 (purple line) when excited at their MLCT region under the same slit width condition (excitation: 10 nm and emission: 10 nm).

**Total reflectance of devices with Ir(III) complexes layers**


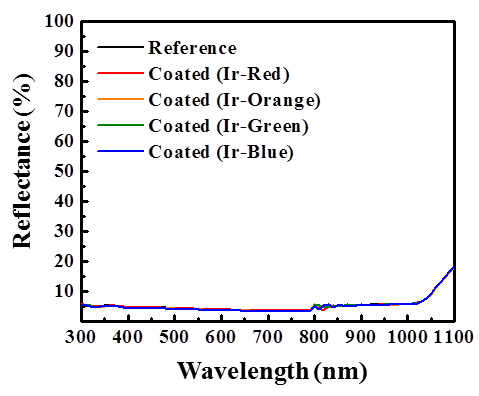


**Supplementary Figure 10. Reflectance of Ir(III) complexes on c-Si NWSCs.** Reflectance spectra of devices employing Ir-Red (red line), Ir-Orange (orange line), Ir-Green (green line), and Ir-Blue (blue line) and an uncoated reference device (black line).

**2D GIWAXD for Ir(III) complexes by USD and spin coating methods**

**
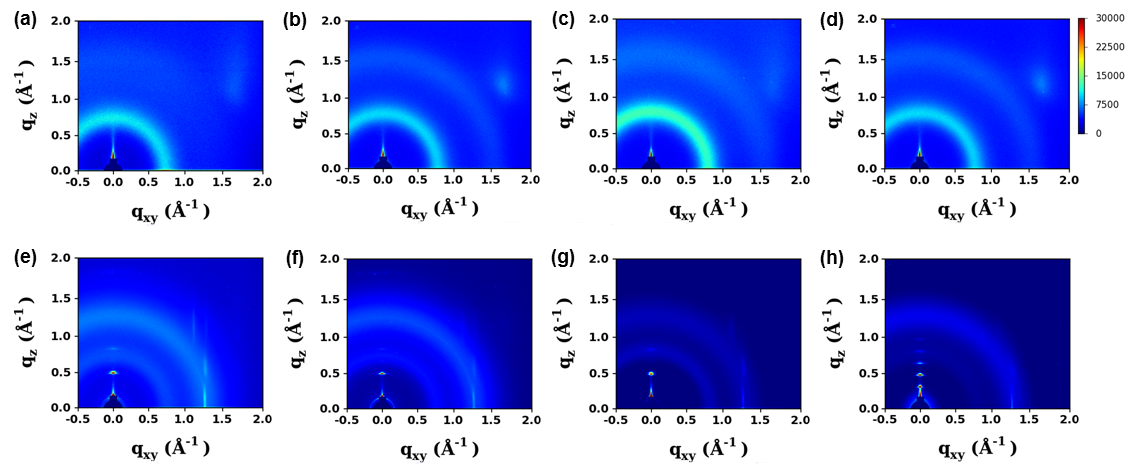
**

**Supplementary Figure 11. 2D GIWAXD patterns of Ir(III) complexes films by USD and spin coating methods. a-h,** 2D GIWAXD patterns of (a) Ir-Red, (b) Ir-Orange, (c) Ir-Green, and (d) Ir-Blue by conventional spin coating methods, and (e) Ir-Red, (f) Ir-Orange, (g) Ir-Green, and (h) Ir-Blue by USD process.

**Micro droplet size of USD calculation**

The USD technique facilitates the micro droplet size by high ultrasonic energy (180 kHz). We could calculated the micro droplet size of Ir(III) complexes solutions in chloroform (CF) by the Sauter mean diameter (SMD) which is the diameter of a sphere that has the same volume/surface area ratio as a particle of interest. The SMD is the average particle size in fluid dynamics and is larger than the median droplet size.^4^

$$\boldsymbol{d}\mathbf{=0.34}\left( \frac{\boldsymbol{8}\boldsymbol{\pi\times s}}{\boldsymbol{p\times}\boldsymbol{f}^{\boldsymbol{2}}} \right)^{\frac{\boldsymbol{1}}{\boldsymbol{3}}}$$

Where *d* is the SMD, s is the surface tension of solvent (kg sec^-2^), p is the density of solvent (kg m^-3^), and f is the frequency of ultrasonic energy (180 kHz).

$$\boldsymbol{d}_{\boldsymbol{CF}}\mathbf{=0.34}\left( \frac{\boldsymbol{8}\boldsymbol{\pi\times0.0266}\left( \boldsymbol{kg}\boldsymbol{sec}^{\boldsymbol{-2}} \right)}{\boldsymbol{1489}\left( \boldsymbol{kg}\boldsymbol{m}^{\boldsymbol{-3}} \right)\boldsymbol{\times}\left( \boldsymbol{180000}\boldsymbol{sec}^{\boldsymbol{-1}} \right)^{\boldsymbol{2}}} \right)^{\frac{\boldsymbol{1}}{\boldsymbol{3}}}\boldsymbol{=8.16\times}\boldsymbol{10}^{\boldsymbol{-6}}\boldsymbol{m=8.2 \mu m}$$

The calculated SMDs of deposited droplets are 8.2 $\mu m$, of which Ir(III) complexes in by USD technique. These micro-sized droplets facilitate the very uniform fine films with high crystallinity.

**Steady state PL for Ir(III) complexes by USD and spin coating methods**

**
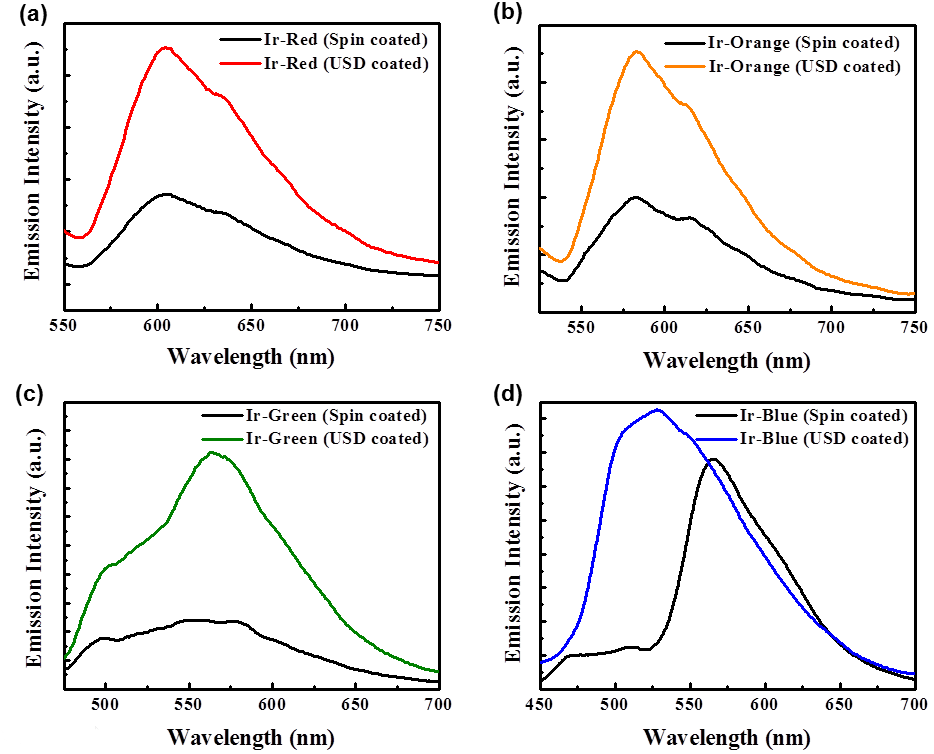
**

**Supplementary Figure 12. PL spectra of Ir(III) complexes films by USD and spin coating methods. a-d,** Steady state PL spectra of (a) Ir-Red, (b) Ir-Orange, (c) Ir-Green, and (d) Ir-Blue by conventional spin coating methods (black lines), and (a) Ir-Red, (b) Ir-Orange, (c) Ir-Green, and (d) Ir-Blue by USD process, red, orange, green, and blue lines, respectively.

**Device performances of c-Si nanowire solar cells with spin coated Ir(III) complexes**


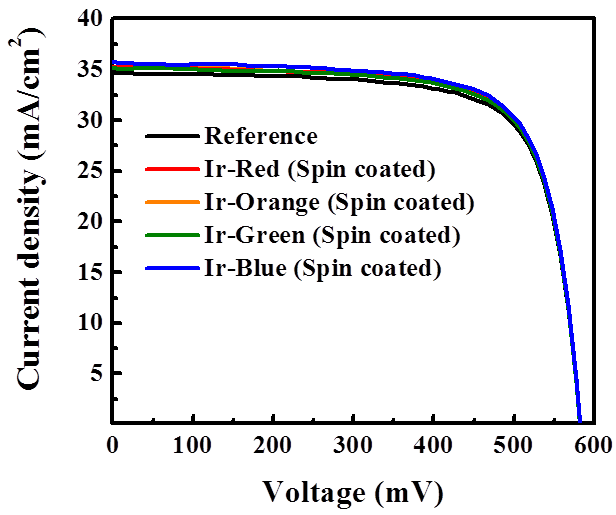


**Supplementary Figure 13. Device performances.** J-V curves of the c-Si nanowire devices with spin coated Ir(III) complexes.

**Reflectance and transmittance spectra of devices with Ir(III) complexes layers**


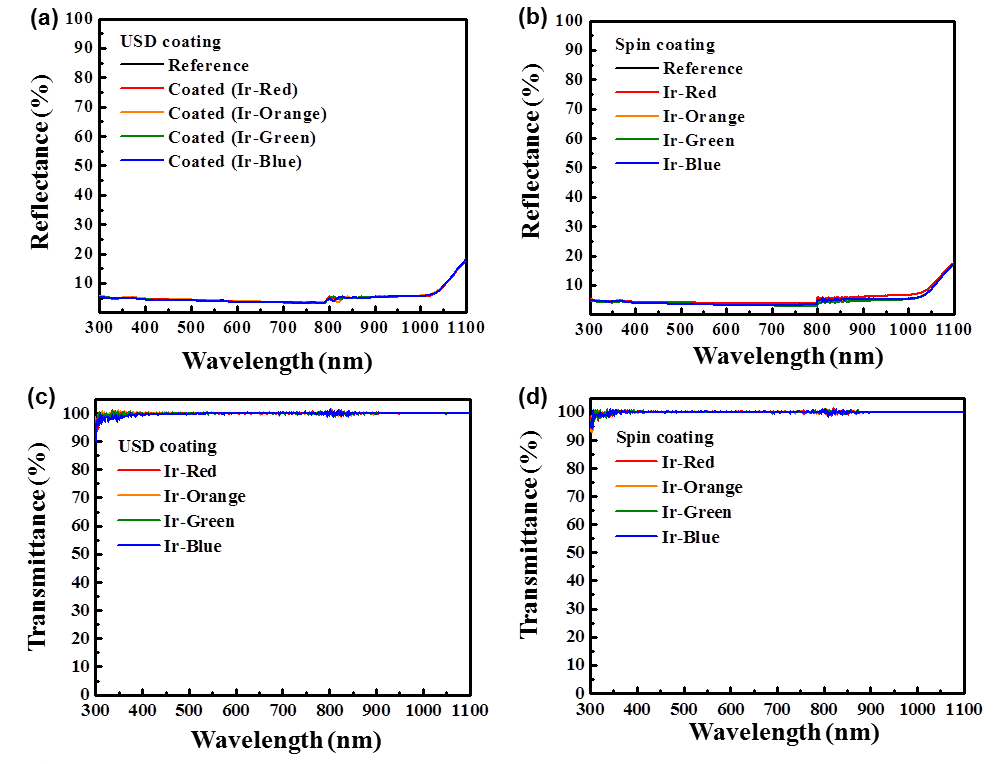


**Supplementary Figure 14. Reflectance and transmittance.** Reflectance spectra of devices with Ir(III) complex layers deposited by USD coating (a) and spin coating (b), Ir-Red (red line), Ir-Orange (orange line), Ir-Green (green line), Ir-Blue (blue line) and an uncoated reference device (black line). Transmittance spectra of the Ir(III) complex layers on glass; Ir-Red (red line), Ir-Orange (orange line), Ir-Green (green line), and Ir-Blue (blue line), by USD coating (c) and spin coating (d).

**SEM images of spin coated and USD coated Ir-Orange on c-Si nanowire substrate**


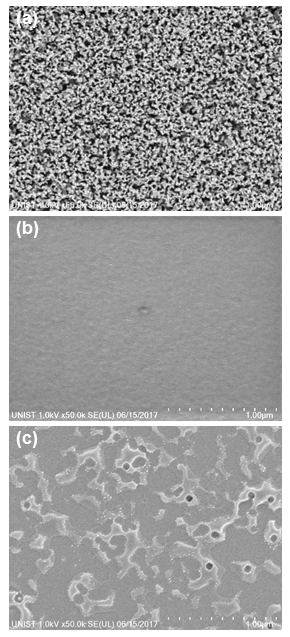


**Supplementary Figure 15. SEM images of Ir-Orange films on c-Si nanowire substrate by USD and spin coating methods. a-c,** top view SEM images of (a) pristine c-Si nanowire substrate, (b) Ir-Orange film by USD, (c) Ir-Orange by conventional spin coating methods.

**Steady-state PL measurement**


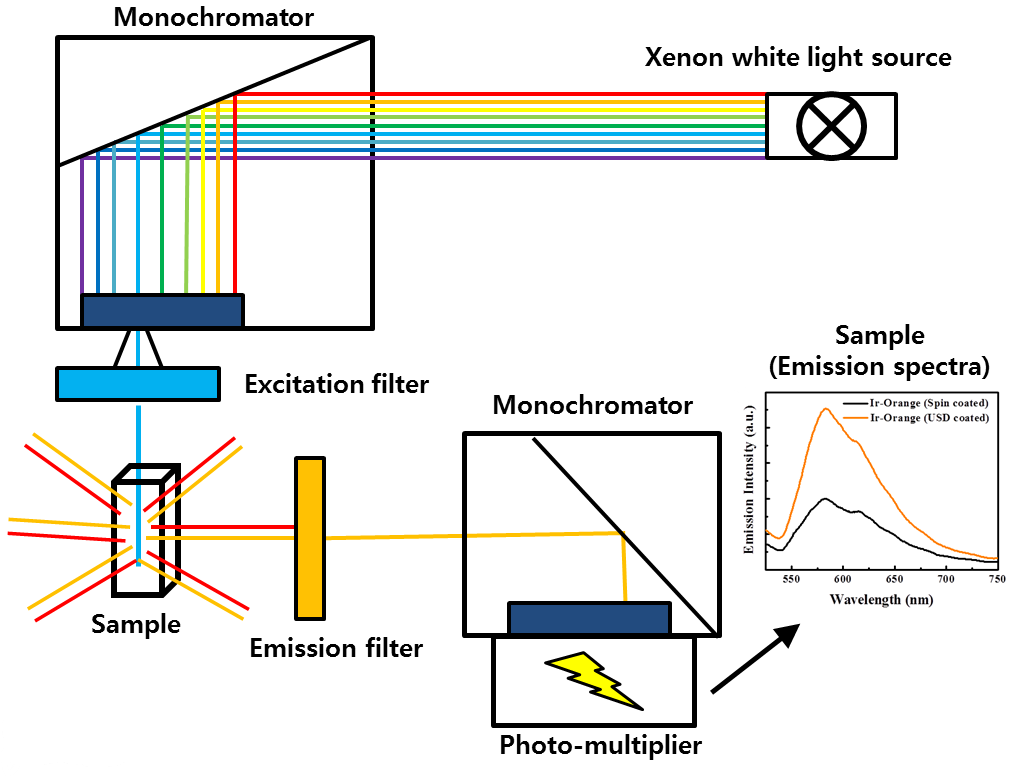


**Supplementary Figure 16. Schematic description of a fluorescence spectrophotometer for steady-state PL measurement.**

A fluorescence spectrophotometer is an analytical instrument used to measure and record the fluorescence of a sample. While recording the fluorescence, the excitation and emission of both wavelengths are scanned.^5^ Spectrometers use a Xenon white light source and a monochromator selects the wavelength of the excitation region. Emission from the target molecule is filtered by an emission filter and then detected by a photo-multiplier (Figure S16). Using the spectrofluorometer (Varian Cary Eclipse), we measured the steady-state PL of the Ir(III) complexes when the excitation wavelength was in their MLCT region (Figure 2a).

**Transient PL measurement**


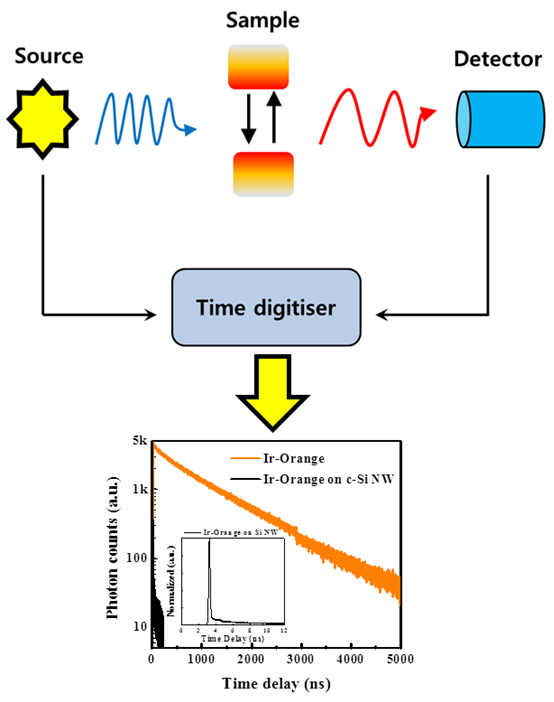


**Supplementary Figure 17. Schematic description of time-correlated single photon counting (TCSPC) for transient PL measurement.**

The principle of TCSPC is the detection of single photons and the measurement of their arrival times in respect to a reference signal, typically the light source (Figure S17). TCSPC is a statistical method requiring a highly repetitive light source to accumulate a sufficient number of photon events for the required statistical data precision.^6^ As the process of capturing a single photon is repeated several thousand or even a million times per second, a sufficiently high number of single photons is processed for the resulting photoluminescence lifetime measurement. Using TCSPC with a Ti:sapphire laser source, we measured the lifetimes of the Ir(III) complexes (Figure 2b).

**Supplementary Table 1.** Summary of device performances for 8 days

|  | Parameter | **0hr** | **24hr** | **48hr** | **72hr** | **96hr** | **120hr** | **144hr** | **168hr** | **192hr** |
| --- | --- | --- | --- | --- | --- | --- | --- | --- | --- | --- |
| **Ir-Orange** | V_oc_(mV) | 587.9 | 598.0 | 587.9 | 587.9 | 587.9 | 587.9 | 587.9 | 587.9 | 587.9 |
|  | J_sc_(mA/cm^2^) | 36.3 | 36.4 | 36.4 | 36.5 | 36.4 | 36.4 | 36.3 | 36.3 | 36.5 |
|  | FF(%) | 77.1 | 77.8 | 78.5 | 77.8 | 77.5 | 77.5 | 77.0 | 77.0 | 77.7 |
|  | PCE(%) | 16.5 | 16.9 | 16.8 | 16.7 | 16.6 | 16.6 | 16.4 | 16.4 | 16.7 |
| **Ir-Red** | V_oc_(mV) | 587.9 | 598.0 | 587.9 | 587.9 | 577.9 | 577.9 | 577.9 | 587.9 | 577.9 |
|  | J_sc_(mA/cm^2^) | 36.2 | 36.1 | 36.3 | 36.2 | 36.3 | 36.3 | 36.3 | 36.2 | 36.3 |
|  | FF(%) | 76.3 | 77.7 | 77.5 | 76.3 | 77.8 | 77.3 | 77.3 | 76.3 | 77.8 |
|  | PCE(%) | 16.3 | 16.8 | 16.5 | 16.3 | 16.3 | 16.2 | 16.2 | 16.3 | 16.3 |
| **Ir-Green** | V_oc_(mV) | 587.9 | 587.9 | 587.9 | 577.9 | 577.9 | 587.9 | 577.9 | 577.9 | 577.9 |
|  | J_sc_(mA/cm^2^) | 36.0 | 35.9 | 35.9 | 35.8 | 35.8 | 35.8 | 35.9 | 36.0 | 36.0 |
|  | FF(%) | 78.2 | 76.9 | 77.1 | 77.3 | 77.2 | 77.5 | 75.8 | 76.7 | 76.0 |
|  | PCE(%) | 16.5 | 16.2 | 16.3 | 16.0 | 16.0 | 16.3 | 15.7 | 16.0 | 15.8 |
| **Ir-Blue** | V_oc_(mV) | 587.9 | 577.9 | 577.9 | 587.9 | 587.9 | 577.9 | 577.9 | 577.9 | 577.9 |
|  | J_sc_(mA/cm^2^) | 36.0 | 35.9 | 36.0 | 35.9 | 36.0 | 36.0 | 36.0 | 35.8 | 36.0 |
|  | FF(%) | 77.0 | 78.2 | 77.9 | 78.0 | 78.2 | 75.9 | 76.3 | 76.1 | 75.9 |
|  | PCE(%) | 16.3 | 16.3 | 16.2 | 16.5 | 16.6 | 15.8 | 15.8 | 15.8 | 15.8 |

**Supplementary Table 2**. Average photovoltaic performances of c-Si nanowire solar cells with spin coated Ir(III) complexes.

| Materials | *J_SC_* (mA/cm^2^) | *V_OC_* (mV) | FF (%) | Efficiency (%) |
| --- | --- | --- | --- | --- |
| Reference | 34.6±0.2 | 578±0.1 | 74.4±0.5 | 14.9±0.1 |
| Orange-OH (spin coated) | 35.2±0.3 | 578±0.1 | 74.4±0.6 | 15.1±0.1 |
| Red-OH (spin coated) | 35.2±0.1 | 578±0.1 | 74.2±0.8 | 15.1±0.1 |
| Green-OH (spin coated) | 35.1±0.1 | 578±0.1 | 74.3±0.3 | 15.1±0.1 |
| Blue-OH (spin coated) | 35.7±0.2 | 578±0.1 | 74.0±0.5 | 15.2±0.1 |

*^a^*The parameters given are averages obtained from four replicate devices for each material.

**Supplementary References**

1 M. Nonoyama, Benzo[*h*]quinolin-10-yl-*N* Iridium(III) complexes, *Bull. Chem. Soc. Jpn*. **47**, 767-768 (1974).

2 T.-H. Kwon, Y. H. Oh, I.-S. Shin, J.-I. Hong, New Approach Toward Fast Response Light-Emitting Electrochemical Cells Based on Neutral Iridium Complexes via Cation Transport, *Adv. Funct. Mater.* **19**, 711-717 (2009).

3 H.-T. Kim, J. H. Seo, J. H. Ahn, M.-J. Baek, H.-D. Um, S. Lee, D.-H. Roh, J.-H. Yum, T. J. Shin, K. Seo, T.-H. Kwon, Customized Energy Down-Shift Using Iridium Complexes for Enhanced Performance of Polymer Solar Cells. *ACS Energy Lett.* **1**, 991-999 (2016).

4. Kim, H. T.; Shin, H.; Jeon, I. Y.; Yousaf, M.; Baik, J.; Cheong, H. W.; Park, N.; Baek, J. B.; Kwon, T. H., Carbon-Heteroatom Bond Formation by an Ultrasonic Chemical Reaction for Energy Storage Systems. *Adv. Mater.* **29**, 1702747-55 (2017).

5. Bhosale, J. S.; Moore, J. E.; Wang, X.; Bermel, P.; Lundstrom, M. S., Steady-state photoluminescent excitation characterization of semiconductor carrier recombination. *Rev. Sci. Instrum.* **87**, 13104-7 (2016).

6. Peronio, P.; Acconcia, G.; Rech, I.; Ghioni, M., Improving the counting efficiency in time-correlated single photon counting experiments by dead-time optimization. *Rev. Sci. Instrum.* **86** , 113101-8 (2015).
